# Supplementary material for: Cerebrospinal fluid proteome maps detect pathogen-specific host response patterns in meningitis
Source: eLife. 2021 Apr 6;10:e64159. doi: 10.7554/eLife.64159 (PMC8043743; doi:10.7554/eLife.64159)
Supplement: Transparent reporting form [file elife-64159-transrepform.docx]

***eLife’s* transparent reporting form**

We encourage authors to provide detailed information *within their submission* to facilitate the interpretation and replication of experiments. Authors can upload supporting documentation to indicate the use of appropriate reporting guidelines for health-related research (see [EQUATOR Network](http://www.equator-network.org/%20)), life science research (see the [BioSharing Information Resource](https://biosharing.org/)), or the [ARRIVE guidelines](http://www.plosbiology.org/article/info:doi/10.1371/journal.pbio.1000412) for reporting work involving animal research. Where applicable, authors should refer to any relevant reporting standards documents in this form.

If you have any questions, please consult our Journal Policies and/or contact us: [editorial@elifesciences.org](mailto:editorial@elifesciences.org).

**Sample-size estimation**

- You should state whether an appropriate sample size was computed when the study was being designed
- You should state the statistical method of sample size computation and any required assumptions
- If no explicit power analysis was used, you should describe how you decided what sample (replicate) size (number) to use

Please outline where this information can be found within the submission (e.g., sections or figure legends), or explain why this information doesn’t apply to your submission:

The samples included in this study are based on two different cohorts of patients enrolled in a prospective study at the Clinic for Infectious Diseases, Lund University Hospital, Lund, Sweden, between March 2006 and November 2009 (Linder et al, 2011). Here, patients with a clinical suspicion of meningitis underwent a lumbar puncture and CSF samples were collected. From this prospective study, a total of 171 CSF samples from 139 patients were included in this study. Consequently, we did not use an explicit power analysis in this study as i) the samples were already collected and 2) no study like the one shown in our manuscript has been performed before.

The details about statistical methods used are described in Figure legends: 2-4, and in Material and Methods, section "Statistical analysis".

**Replicates**

- You should report how often each experiment was performed
- You should include a definition of biological versus technical replication
- The data obtained should be provided and sufficient information should be provided to indicate the number of independent biological and/or technical replicates
- If you encountered any outliers, you should describe how these were handled
- Criteria for exclusion/inclusion of data should be clearly stated
- High-throughput sequence data should be uploaded before submission, with a private link for reviewers provided (these are available from both GEO and ArrayExpress)

Please outline where this information can be found within the submission (e.g., sections or figure legends), or explain why this information doesn’t apply to your submission:

Each cerebrospinal fluid (CSF) sample was considered as one biological replicate and analyzed separately with LC-MS/MS.

We have indicated the number of independent biological replicates in Materials and Methods, section "Patients and CSF samples", as well as in Results, section "Construction of a compendium of SWATH-MS CSF proteome maps from meningitis patients".

No technical replicates were used in this study.

The mass spectrometry proteomics data have been deposited to the ProteomeXchange Consortium via the PRIDE partner repository with the dataset identifier PXD023174.

No outliers were encountered in this study.

The acquired data was manually curated to remove immunoglobulin variable chain proteins, as stated in the headers and legends of Supplementary tables 2 and 3.

**Statistical reporting**

- Statistical analysis methods should be described and justified
- Raw data should be presented in figures whenever informative to do so (typically when N per group is less than 10)
- For each experiment, you should identify the statistical tests used, exact values of N, definitions of center, methods of multiple test correction, and dispersion and precision measures (e.g., mean, median, SD, SEM, confidence intervals; and, for the major substantive results, a measure of effect size (e.g., Pearson's r, Cohen's d)
- Report exact p-values wherever possible alongside the summary statistics and 95% confidence intervals. These should be reported for all key questions and not only when the p-value is less than 0.05.

Please outline where this information can be found within the submission (e.g., sections or figure legends), or explain why this information doesn’t apply to your submission:

For statistical analyses, a Benjamini-Hochberg corrected t-test was used. The details of each statistical method, such as dispersion and precision measures, p-values and confidence intervals are visualized in each figure and further described within the figure legends of the respective figure (Figure legends: 1-4).

For lasso regression analysis, detailed statistical description can be found in Materials and Methods, section "Statistical analysis".

Exact values of N are always referred to throughout the whole manuscript.

(For large datasets, or papers with a very large number of statistical tests, you may upload a single table file with tests, Ns, etc., with reference to sections in the manuscript.)

**Group allocation**

- Indicate how samples were allocated into experimental groups (in the case of clinical studies, please specify allocation to treatment method); if randomization was used, please also state if restricted randomization was applied
- Indicate if masking was used during group allocation, data collection and/or data analysis

Please outline where this information can be found within the submission (e.g., sections or figure legends), or explain why this information doesn’t apply to your submission:

The CSF samples were allocated to each experimental group based on diagnostic information received from the responsible clinician.

For data analysis, only samples with confirmed microbiological diagnosis to species level were included in our study.

Patients suspected of suffering from meningitis but showed no elevated clinical parameters and were further declared healthy were included as control samples.

Detailed description of how CSF samples were categorized in each experimental group can be found in Material and Methods, section: "Patients and CSF samples".

**Additional data files (“source data”)**

- We encourage you to upload relevant additional data files, such as numerical data that are represented as a graph in a figure, or as a summary table
- Where provided, these should be in the most useful format, and they can be uploaded as “Source data” files linked to a main figure or table
- Include model definition files including the full list of parameters used
- Include code used for data analysis (e.g., R, MatLab)
- Avoid stating that data files are “available upon request”

Please indicate the figures or tables for which source data files have been provided:

The mass spectrometry proteomics data have been deposited to the ProteomeXchange Consortium via the PRIDE partner repository with the dataset identifier PXD023174.
